# The Rarity in the Rarity: Presentation of Two Rare and Unusual Cases of Nodular Fasciitis and Proliferative Fasciitis

**DOI:** 10.7759/cureus.63357

**Published:** 2024-06-28

**Authors:** Giulia Bagaloni, Anna Colagrande, Giuseppe Ingravallo, Andrea Marzullo, Gerardo Cazzato

**Affiliations:** 1 Section of Molecular Pathology, Department of Precision and Regenerative Medicine and Ionian Area (DiMePRe-J), University of Bari Aldo Moro, Bari, ITA; 2 Section of Pathology, Department of Emergency and Organ Transplantation, University of Bari Aldo Moro, Bari, ITA

**Keywords:** rarity, vulva, children, proliferative fasciitis, nodular fasciitis

## Abstract

Nodular fasciitis (NF) and proliferative fasciitis (PF) are benign, reactive mesenchymal neoplasms that can mimic malignancies due to their rapid growth and histological characteristics. NF typically affects the subcutaneous tissue, occasionally involving muscles and fascia, predominantly in young adults, and appears frequently in the upper extremities, trunk, and head/neck. PF, a pseudosarcomatous lesion, primarily occurs in the subcutaneous tissue of adults aged 40-70 years and is uncommon in younger populations. This article presents two pediatric cases of NF and PF in unusual locations: a six-year-old girl with a vulvar NF and a 10-year-old girl with a gluteal PF. Both cases demonstrated rapid growth and distinct histological features, confirmed by immunohistochemical analyses and fluorescence in situ hybridization (FISH). These cases underscore the importance of accurate histological recognition to avoid misdiagnosis and ensure appropriate treatment, highlighting the rarity of such occurrences in children and the need for awareness among clinicians and pathologists.

## Introduction

Nodular fasciitis (NF) and proliferative fasciitis (PF) are reactive, mesenchymal neoplasms that can present diagnostic challenges due to their rapid growth and histological features that can mimic malignancy [1-2]. NF is a self-limiting fibroblastic/myofibroblastic proliferation usually affecting subcutaneous tissue and sometimes muscles and fascia. It usually arises in the upper extremities, often in the flexor aspect of the forearms, trunk, and head/neck [3]. On the basis of its clinical and histological features, a fast-growing solitary tumor (that usually worries the patient) with high cellularity, brisk mitotic count, and positivity for actin, NF is considered to be a benign mimic of sarcoma. It is a relatively common mesenchymal neoplasm that predominates in young adults, although it can occur at virtually any age, and it has no significant sex predilection [4]. PF is a solid pseudo-sarcomatous subcutaneous lesion of the soft tissue, and for definition, it is distinct from proliferative myositis (PM) that occurs only in the muscle [5]. The presence of plump myofibroblastic/fibroblastic spindle cells, the density of large ganglion-like cells (modified fibroblasts), and common mitotic figures are the distinctive features of PF and often grow along fibrous connective tissue septa; most lesions occur in the fascia and subcutaneous tissue of the extremities, with predominance in the upper extremity [2,5]. It usually occurs in adults aged between 40 and 70 years and is very uncommon in children and adolescents [5].

In this article, we want to show two cases of unusual locations of NF and PF in childhood, underscoring the importance of correct histological recognition to avoid misdiagnosis that can potentially affect the course of the therapy.

## Case presentation

Case 1

A six-year-old girl went to the Pediatric Surgery Unit in May 2023 (not a consecutive case) for the appearance of a perineal neoformation in the vulvar region at five months old, which had worried her parents and which had grown so quickly, requiring the attention of a physician. After surgical excision, the sample was sent to the Complex Pathology Unit.

**Figure 1:**
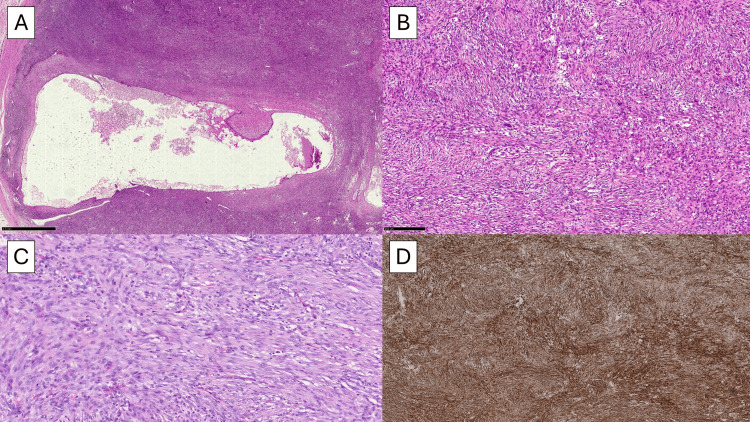
(A) Histopathological photomicrograph showing plump, spindle-shaped cells with degenerative central cystic phenomena (H&E, original magnification 4x). (B) Histological photomicrograph showing a storiform pattern with S-shaped and/or C-shaped fascicles and little collagen (H&E, original magnification 20x). (C) Another field of the previous picture showing also some extravasated eritrocytes between the fibroblastic proliferation (H&E, original magnification 40x). (D) Immunohistochemical preparation for SMA: note the diffuse positivity of the cells in the typical tram-tracking pattern (H&E, original magnification 10x). H&E: hematoxylin and eosin, SMA: smooth muscle actin

Macroscopically, the lesion measured 3.5 x 2.5 x 2.0 cm and was described as centrally colliquated. After preparation of the sections in hematoxylin-eosin (HE), a cellular proliferation of plump spindle cells was observed, without significant atypia and/or pleomorphism and with some typical mitotic figures (Figure 1A). In some fields, it was possible to appreciate a partial discohesive and myxoid pattern, with tissue culture-like features (Figure 1B). In other fields, there was a storiform pattern and centrally was present a large cystic component; finally, there were extravasated erythrocytes, lymphocytes, and rarely some osteoclast-like giant cells (Figure 1C). The borders were well-defined with a focal infiltrative pattern. From an immunohistochemical point of view, the lesion was strongly positive for smooth muscle actin (SMA) in the typical tram-track pattern (Figure 1D) and vimentin and negative for desmin, CK-pool, p53, estrogen receptor (ER), and CD34. CD68 and CD163 were positive in the histiocytic accompanying cell population. The proliferation fraction evaluated with Ki67+ was unevenly distributed, with hot spots of approximately 7-8%. The histological picture and immunohistochemical characteristics allowed us to conclude the diagnosis as NF. After that, fluorescence in situ hybridization (FISH) confirmed the USP6 rearrangement. At the 12-month follow-up, the patient is currently disease-free.

Case 2

**Figure 2:**
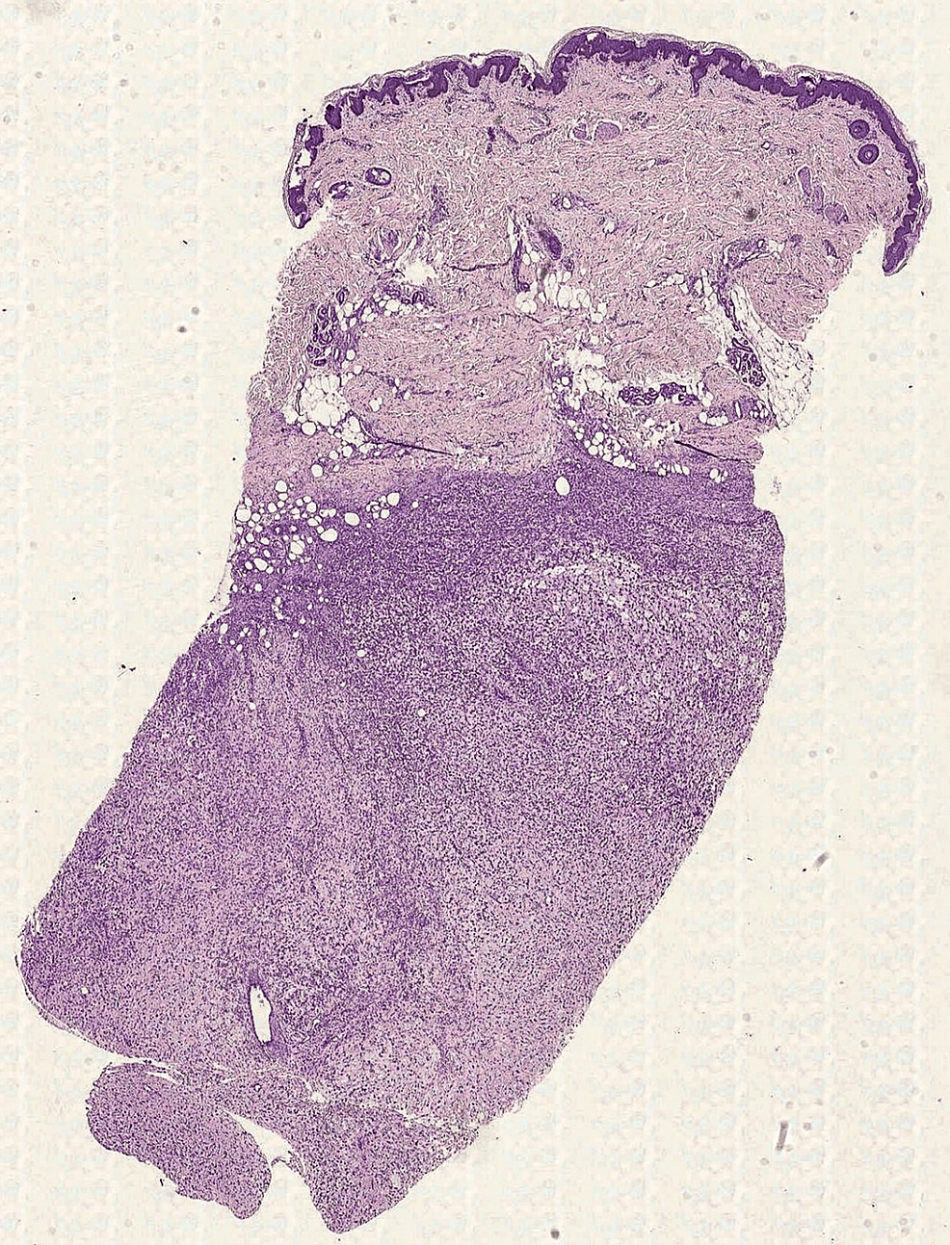
Histological photomicrograph showing an incisional skin biopsy partially occupied by cellular proliferation in the deep dermis/subcutis (H&E, original magnification 2x). H&E: hematoxylin and eosin

**Figure 3:**
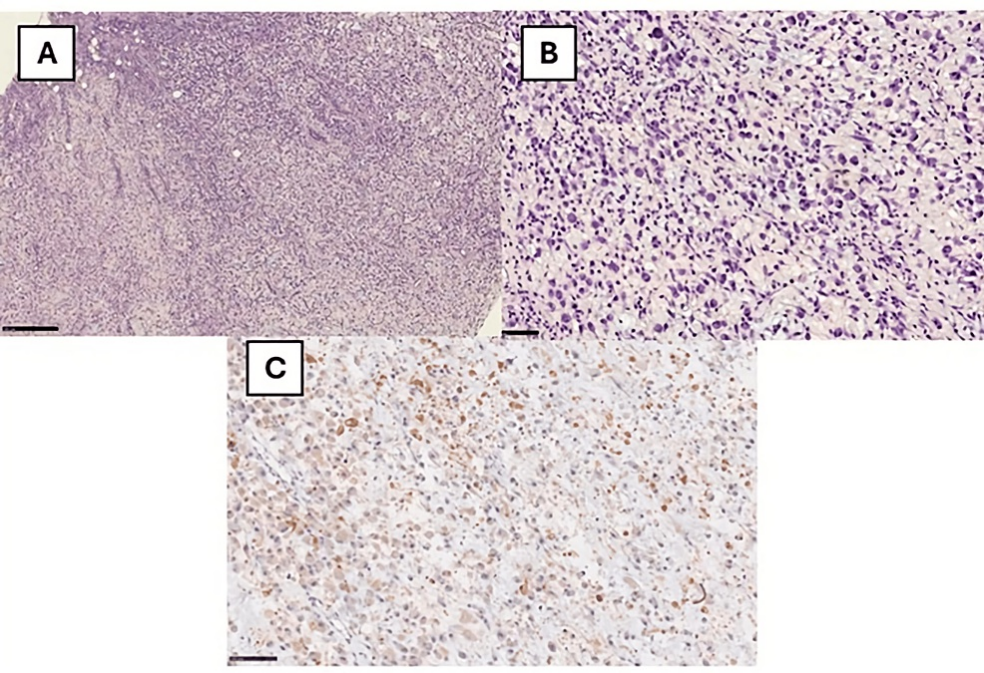
(A) Histopathological photomicrograph showing a pseudosarcomatous cellular proliferation constituted of large cells, with abundant cytoplasm and loose intercalated stroma, in some places myxoid (H&E, original magnification 10x). (B) Histological photomicrograph showing many “ganglion-like” cells, with some multinucleated elements (Sternberg-like) (H&E, original magnification 40x). (C) Immunohistochemical preparation for anti-factor XIII: note the partial positivity of the cells (Immunohistochemistry for anti-factor XIII (original magnification 40x). H&E: hematoxylin and eosin

A 10-year-old girl came to the doctor in April 2022 (not a consecutive case) for observation due to the onset of a lesion in the gluteal region, which cannot be better-dated. We opted for an incisional skin biopsy, which allowed us to appreciate cellular proliferation characterized by the presence of large cells (Figure 2). In particular, the cells were characterized by abundant eosinophilic cytoplasm, with vesicular nuclei and nucleoli close to the edge of the nucleus (rhabdoid-like appearance) and some multinucleated, especially binucleated, Sternberg-like elements (Figure 3B). Furthermore, there was the presence of many “ganglion-like” cells (Figure 3B). The stroma interspersed with the cell population appeared loose in several places, taking on a clear myxoid appearance in some areas (Figure 3A); furthermore, it was associated with a modest amount of mainly chronic inflammation. From an immunohistochemical point of view, there was clear positivity for vimentin, partially for CD68, factor XIII (Figure 3C), SMA, actin HHF-35, and calponin. Staining for melanocyte (Melan-A, HMB-45, SOX10, MiTF, and tyrosinase), epithelial (EMA, cytokeratins), and muscle markers such as desmin and myogenin were negative. INI1 (SMARCB1) was retained, and finally, markers for CD45, ALK, CD34, and factor VIII were negative. The neoplastic proliferation index assessed by Ki67+ was approximately 15%.

Taking into account the histological and immunohistochemical results, a diagnosis of PF was made. After that, the patient underwent enlargement of the surgical exeresis with free margins. At the 24-month follow-up, the patient is currently disease-free.

## Discussion

NF is a self-limiting mesenchymal neoplasm that usually occurs on the surface of the fascia and extends into the subcutaneous tissue [3-4]. Occasional intramuscular cases have been reported, with even rare dermal localization [4]. Clinically, it is characterized by its rapid growth and usually does not reach more than 5 cm in its maximum diameter [4]. From a molecular point of view, MYH9::USP6 represents the most recurrent fusion gene, but there are also other possible fusion patterns [4]. NF in the vulvar/peri-vulvar skin is rare, with only a few cases reported in the literature to the best of our knowledge [6-10]. Interestingly, in a recent paper [11], the authors reported a case of NF in a 52-year-old perimenopausal woman who presented with a vulvar mass.

In this case, desmin, usually negative in this kind of lesions, was strongly positive, and the authors considered this feature as reflective of the potential origin of the lesion from desmin-positive myofibroblastic cells of the vulvar region. In our case, desmin was totally negative although the region seems to be similar. In the same paper, our FISH results were also consistent with NF for the presence of USP6 rearrangement. In terms of differential diagnosis, the most important entities are represented by inflammatory myofibroblastic tumor (IMT) that is ALK-positive in about 50% of cases [11] and also low-grade myofibroblastic sarcoma, which usually is more infiltrative. A recent abstract-type paper [12] presented a case of a 27-year-old female with a history of one-month labial swelling and pain, suspected for an abscess. The morphological, immunohistochemical, and FISH results were similar to ours, but it is worthy of note the potential of other differential diagnoses with cellular angiofibroma, angiomyofibroblastoma, and aggressive angiomyxoma, for which a careful histological evaluation of the myxoid component is mandatory.

Regarding PF, it is important to underscore that usually it occurs in middle-aged and/or older adults, and rarely it was described in children [1], such as in our case. Regarding differential diagnosis, PF has to be distinguished by NF, IMT, and also ganglioneuroblastoma for the presence of ganglion-like cells. Furthermore, an appropriate morphological, immunohistochemical, and, if necessary, molecular approach allows the differential diagnosis (particularly in childhood) with embryonal rhabdomyosarcoma, pleomorphic rhabdomyosarcoma, and undifferentiated pleomorphic sarcoma (UPS) [13].

A recent study examined the FOS gene status in six PF/PM cases (one PM and five PFs). A strong c-FOS expression was seen in epithelioid cells in five adult instances, but spindle cells showed mainly negative expression. The epithelioid cells of these five cases showed FOS gene rearrangement, as demonstrated by FISH. One instance of RNA sequencing revealed a FOS-VIM fusion transcript, which was further validated by Sanger sequencing, VIM FISH, and reverse transcriptase-polymerase chain reaction (RT-PCR). There was no evidence of FOS rearrangement or c-FOS expression in a pediatric PF case. Forty-five different mesenchymal tumor forms, including pleomorphic sarcoma with epithelioid characteristics and epithelioid sarcoma, which may mimic PF/PM, showed negative results for c-FOS immunohistochemistry.

The c-FOS overexpression and recurrent FOS rearrangement in PF/PM indicate that these lesions are cancerous. The epithelioid cells were the primary site of the FOS anomaly, suggesting the presence of two distinct cell types in these lesions. Thus, PF/PM may be accurately distinguished from other cancers that are identical to it using c-FOS immunohistochemistry.

## Conclusions

NF and PF are benign yet rapidly growing mesenchymal neoplasms that can be mistaken for malignant tumors due to their histological features. NF predominantly affects young adults and commonly arises in the subcutaneous tissues of the upper extremities, whereas PF typically occurs in middle-aged to older adults and is rare in children. The two pediatric cases presented in this study, NF in the vulvar region of a six-year-old girl and PF in the gluteal region of a 10-year-old girl, highlight the unusual occurrence of these conditions in childhood. Accurate histological and immunohistochemical evaluation, supported by molecular analysis, is crucial to avoid misdiagnosis and ensure appropriate management. These cases underscore the importance of recognizing the benign nature of these proliferations despite their alarming presentation, thus preventing unnecessary aggressive treatments and guiding proper therapeutic strategies.
